# The Association of Embracing with Daily Mood and General Life Satisfaction: An Ecological Momentary Assessment Study

**DOI:** 10.1007/s10919-022-00411-8

**Published:** 2022-08-04

**Authors:** Julian Packheiser, Imke Marlene Malek, Jacqueline Sophia Reichart, Laura Katona, Maike Luhmann, Sebastian Ocklenburg

**Affiliations:** 1 Social Brain Lab, Netherlands Institute for Neuroscience, Amsterdam, The Netherlands; 2 Faculty of Psychology, Institute of Cognitive Neuroscience, Biopsychology, Ruhr University Bochum, Bochum, Germany; 3 Faculty of Psychology, Ruhr University Bochum, Bochum, Germany; 4 Department of Psychology, Faculty of Human Sciences, Medical School Hamburg, Hamburg, Germany; 5 ICAN Institute for Cognitive and Affective Neuroscience, Medical School Hamburg, Hamburg, Germany

**Keywords:** Embracing, Social touch, Loneliness, Depression, Ecological momentary assessment

## Abstract

Embracing has several positive health effects, such as lowering blood pressure and decreasing infection risk. However, its association with general life satisfaction and daily mood has not been researched in detail. Here, we used a smartphone-based ecological momentary assessment (EMA) approach to monitor the daily number of embraces and daily mood in a sample of 94 adults over the course of seven days. We found that embracing frequency differed slightly over the week, with embracing occurring more frequently on weekends than on weekdays. We also found that higher daily embracing frequencies were associated with better daily mood using multilevel modeling. Only singles benefitted from increases in average embracing regarding their life satisfaction, whereas individuals in a relationship were unaffected by their embracing tendencies. Although our results are strictly correlational and do not indicate any direction or causality, embraces may be important for daily mood and general life satisfaction, but their efficacy seems to depend on relationship status.

**Supplementary Information:**

The online version contains supplementary material available at 10.1007/s10919-022-00411-8.

## Introduction

Social touch is an important means of nonverbal communication and emotion regulation from infancy throughout the whole lifespan (Cascio et al., 2019). Across all cultures, humans engage in touch-based social interactions (Ocklenburg et al., 2018). One of the most common forms of social touch is the act of embracing between two individuals. Despite its prevalence across all humans and its long history (the earliest recorded archeological evidence for a human embrace dates back to Neolithic times, Urbanus, 2008), there is still very little research on how this behavior occurs in daily life. While some information is now available on the duration of human embraces (the average embrace lasts approximately 3 s, Nagy, 2011) and the perception of pleasantness with respect to the duration (5 to 10 s are perceived as more pleasant than a 1 s duration embrace, Dueren et al., 2021) there is currently no study that has quantified the frequency of embraces in everyday life.

In contrast, embracing has been studied extensively in the context of increasing physical health parameters. For example, higher frequencies of embracing have been linked to lower blood pressure and heart rate in premenopausal women (Light et al., 2005). Moreover, embracing reduces the physiological effects of stress. Compared to a no-hug control group, individuals who received a ten-minute period of handholding with their partner followed by a 20 s hug with their partner showed lower systolic and diastolic blood pressures as well as lower heart rate increases following a stressful public speaking task (Grewen et al., 2003). Embracing can furthermore reduce the risk of infection. In an experimental study, volunteers were infected with a virus that caused common-cold-like symptoms and monitored over two weeks with regard to their embracing behavior (Cohen et al., 2015). A higher embracing frequency prior to virus administration was related to a reduced infection risk. The authors suggested that embracing might effectively convey social support, which in turn decreases infection risk. Finally, embracing decreases the presence of proinflammatory cytokines in the body and can buffer against the effects of acute stress, further suggesting a positive health benefit from this form of social touch (Berretz et al., 2022; van Raalte & Floyd, 2020).

While all these studies demonstrate the positive effect of embracing on physical health, less research has been conducted on the impact of embracing on subjective well-being. Subjective well-being is constituted by an individual’s overall life satisfaction and the relative frequency of experienced positive and negative affect (Diener, 1984). Embraces with the romantic partner are behaviorally and neurophysiologically associated with stronger positive affective states compared to embraces of a body pillow (Packheiser et al., 2021). One recent study monitored daily embraces over the course of 14 days and assessed both mood and conflict situations in individuals across this time span (Murphy et al., 2018). They found that embracing generally increased positive mood and decreased negative mood regardless of conflict situations. When confronted with conflict situations, negative mood increased substantially in the participants, but this effect was strongly mitigated if embraces occurred on a given day. Thus, the results of the study by Murphy et al. (2018) demonstrate that embraces also benefit subjective well-being as they increase daily mood. Importantly, however, the study by Murphy et al. (2018) focused on situational factors affecting the efficacy of embraces on mental health, namely in conflict situation, while not including more stable variables that generally affect mood such as loneliness (Frison & Eggermont, 2020).

Loneliness is defined as the subjectively felt discrepancy between the desired and the actual connectedness and intimacy of social relationships (Gierveld and van Tilburg, 2006; Hawkley & Cacioppo, 2010). Loneliness has been demonstrated to be significantly associated both with depressed mood (Zawadzki et al., 2013) and reduced life satisfaction (Goodwin et al., 2001). Whereas there is thus good evidence that loneliness and negative subjective well-being are linked, it is less known if social touch can positively regulate negative affect. One study found that social touch decreases feelings of social exclusion in the cyberball task, a commonly used social exclusion paradigm (Mohr et al., 2017). While social exclusion and loneliness are not the same concepts, this study at least tentatively suggests that embracing as a form of social touch could potentially be associated with a reduction of loneliness and therefore, ultimately increase subjective well-being. Previous studies on social touch have supported this idea as massages by an unknown experimenter can reduce the feeling of loneliness (Heatley Tejada et al., 2020). A recent large-scale study during the COVID-19 pandemic found that especially intimate social touch can be beneficial and alleviate loneliness (Mohr et al., 2021). A study conducted in elderly participants demonstrated that comfort touch improved feelings of life satisfaction as well as subjective well-being overall (Butts, 2001).

In the present study, we used a smartphone-based ecological momentary assessment (EMA) design (Schembre et al., 2018; Shiffman et al., 2008) to investigate the associations between embracing, daily mood, and life satisfaction. The strength of EMA is that daily behaviors can be tracked more accurately as EMA significantly decreases recall bias due to the temporal closeness between events and the data collection (Shiffman et al., 2008). Furthermore, EMA significantly improves the studying of subjective well-being since the structure of the mood dimension of subjective well-being is highly dynamic and can substantially change over short time-spans (Luhmann et al., 2021). However, subjective well-being is also dependent on more stable trait variables such as personality traits, indicating that subjective well-being is a complex construct comprising both highly dynamic state and less dynamic trait components (Luhmann et al., 2021). Using EMA, both state and trait variables can be measured, and their independent contributions as well as interactions can be examined. Another strength of EMA is that behavior is reported regularly and thus more accurately, as it has been shown that behavioral reports from several months ago have poor accuracy (Shiffman et al., 1997).

The aims of the present study were two-fold: first, we aimed to provide accurate descriptive reference data of daily embracing behavior across the population using EMA as there is almost no data on the occurrence of this form of social touch in the current literature. Second, we aimed to investigate the relationship between momentary mood and overall life satisfaction and daily embracing. To this end, participants were asked to report the number of embraces, the number of embraced individuals, and their mood for seven consecutive days. At the end of the seven-day period, participants further reported a general assessment on their life satisfaction. We also assessed personality traits that could affect embracing behavior as well as mood and life satisfaction. Here, we assessed the Big Five personality traits as social touch, mood, as well as life satisfaction have been shown to positively correlate with the Extraversion domain of the Big Five (Anglim et al., 2020; DeNeve & Cooper, 1998; Larsen & Ketelaar, 1989; Rusting & Larsen, 1997; Schimmack et al., 2004; Trotter et al., 2018), whereas Neuroticism has been negatively associated with mood and life satisfaction (Larsen & Ketelaar, 1989; Rusting & Larsen, 1997; Schimmack et al., 2004). Moreover, we assessed relationship status, as both romantic and social touch are potentially increased in individuals in stable long-term relationships (Triscoli et al., 2017). Furthermore, it has been shown that being in a relationship is generally associated with higher subjective well-being (Dush & Amato, 2005).

Since the first part of the study aimed to gather descriptive reference data, no specific confirmatory hypotheses were formulated. For the second part of the study, we hypothesized that daily mood and life satisfaction are positively associated with daily or average embracing frequency, respectively. In accordance with previous findings, we also predicted that loneliness and Neuroticism are negatively associated with mood and life satisfaction, whereas Extraversion and being in a relationship are positively associated with these variables. In exploratory analyses, we also investigated if more stable characteristics such as feelings of loneliness or personality traits possibly moderate the association between embracing and well-being.

## Method

### Participants

The final sample after applying the exclusion criteria included 94 adult individuals with an average age of 26.36 years (*SD* = 10.93; range: 18 to 69 years). Participants were excluded if they reported mental or neurological disorders in the demographic questionnaire or if they failed to comply with the procedural demands to report to the daily questionnaires. No participant had to be excluded based on the former criterion and one participant had to be excluded based on the latter criterion. A sensitivity analysis was conducted using g*Power 3.1.9.7 (Faul et al., 2007). To reach 80% power with our sample size in a multiple linear regression model with five tested predictors, an effect size of *Cohen’s f*^*2*^ = 0.15 would have been needed which is a medium to large effect. Thus, based on the effects found by Murphy et al. (2018) who found large effects of embracing on positive and negative mood changes in same-day conflict situations (Cohen’s *d* = 0.84), the sample size should have been sufficient to detect an effect reliably. In the present study, 42 participants were male and 52 were female. Fifty-nine participants were in a romantic long-term relationship and 35 were single. The average relationship duration was 98.63 months (*SD* = 140.60). All participants were native German speakers. The study was approved by the local ethics committee at the faculty of psychology at Ruhr University Bochum. All participants gave written informed consent and were treated in accordance with the declaration of Helsinki.

### Ecological Momentary Assessment

Data collection took place from April to July 2019 and thus prior to the COVID-19 pandemic. Participants were tested using an ecological momentary assessment consisting of seven subsequent test sessions (individual assessment weeks differed between participants and could start on any weekday). On each of the seven days of the week, participants received a short questionnaire via a Qualtrics link (https://www.qualtrics.com/de/) that was sent to them via email in the evening.

### Measures

On each day, participants were asked to indicate which day of the week it was, how often they had embraced someone in the last 24 h and how many different individuals they had embraced in the last 24 h. No specific instructions were given as to what counts as an embrace to more readily assess this at the end of the day as this can be a rather individual behavior. Furthermore, participants were asked the question: “How do you rate your today’s mood?” to assess their daily mood. The question was asked using a 5-point Likert scale from -2 (*bad*) to 2 (*good*). These one-dimensional mood scales have been validated in clinical research for example in the prediction of depression relapse (van Rijsbergen et al., 2012) and facilitate the data collection process and increase compliance when mood has to be assessed regularly. After the end of the seven-day EMA segment, participants were tested with several established measures to assess variables that might be related to embracing. Specifically, loneliness was assessed using the German version of the UCLA Loneliness Scale (Version 3) (Döring & Bortz, 1993; Russell, 1996). This questionnaire consists of 20 items that need to be answered on a four-point scale from “*never*” to “*often*”. Life satisfaction was measured via the German version of the satisfaction with life scale (SWLS, Glaesmer et al., 2011). Here, five items asked about the participants’ life satisfaction on a seven-point Likert scale ranging from “*strongly disagree*” to “*strongly agree*”. Since momentary mood and life satisfaction have been demonstrated to be modulated by the Extraversion and Neuroticism domains of the Big Five personality traits (Andersen & Leibowitz, 1978; Anglim et al., 2020; Triscoli et al., 2017; Trotter et al., 2018), we also assessed them using the German version of the extra-short form of the Big Five Inventory–2 (BFI-2-XS) (Rammstedt et al., 2018; Soto & John, 2017). This questionnaire contains 15 items that have to be answered on a five-point Likert scale from “*disagree strongly*” to “*agree strongly*”. It retains much of the full BFI-2’s reliability and validity for assessment of the Big Five personality domains (Soto & John, 2017).

### Statistical Analyses

Statistical analyses were conducted using R 4.1.0 and IBM SPSS Statistics 21. In a first step, we analyzed differences in embracing behavior and mood across the week. A repeated measures ANOVA was conducted to determine if these variables were associated with a given day of the week.

To test our hypotheses regarding daily mood, we used multilevel modeling with daily mood as the dependent variable and daily embracing, personal traits, and feelings of loneliness as independent variables. First, a base model without any interactions was tested. Here, daily embraces were used as level 1 predictor and loneliness, relationship status (relationship = 0 vs. single = 1), Extraversion, and Neuroticism as level 2 predictors. Daily embraces as level 1 predictor were centered on the person mean. Multicollinearity between the predictors of the base model was checked using the Variance Inflation Factor (VIF, see Supplementary Table 1).

The estimation of cross-level interactions between level 1 and level 2 predictors followed the guidelines outlined in Aguinis et al. (2013). As the cross-level interactions were exploratory and not hypothesis-driven, only the post-data collection guidelines were adhered to. (1) The level 1 predictor variable was again centered on the person mean, (2) cross-level interactions were illustrated, and (3) the level 2 predictor variable was used as moderator. (4) Since we did not hypothesize any cross-level interactions of daily embracing with any level 2 predictor, we ran four successive models and computed cross-level interactions between daily embracing and loneliness, relationship status, Neuroticism, and Extraversion in a further analysis. Interactions were estimated in separate models as opposed to in one composite model for statistical reasons. An assessment of variance inflation factors of a model containing all interactions simultaneously revealed strong increases in VIFs as opposed to models with only one interaction term (see Supplementary Table 2). Since high VIFs result in decreased reliability of the model, each interaction that included one of the four level 2 predictors was assessed individually in separate models. To account for the possibility of alpha error accumulation in these exploratory models, we decided to apply a Bonferroni correction for these separate models. As we tested four different cross-level interactions, we used a Bonferroni-corrected statistical threshold (α = 5%/4 or 1.25%) to test for significance. (5) No higher-level interactions were investigated due to already inflated VIFs in assessing simultaneous two-way interactions. (6) Effect sizes of cross-level interactions were included using the Pseudo *R*^2^ of each model and (7) all coefficients, standard errors and confidence intervals for each predictor were reported in detail.

To test our hypotheses regarding life satisfaction, we ran a single level linear model using life satisfaction as dependent variable. Instead of daily embraces, we used the average number of daily embraces across the week as predictor, since this variable indicates the general tendency to embrace overall. As for daily mood, we included loneliness, relationship status, Extraversion, and Neuroticism into the model. Since relationship status represented a categorical variable, we report differences between singles and individuals in a relationship in Supplementary Table 3. Multicollinearity between the predictors of the base model was again tested using the VIF (see Supplementary Table 4).

Interactions between average embracing and the other level 2 predictors followed the same principle as for the analysis of daily embracing outlined above. As before, we did not hypothesize any interactions with average embracing and therefore explored interactions between embracing and loneliness, relationship status, Neuroticism, and Extraversion. Identically to the analysis for daily embracing, VIFs were considerably higher if all interactions were included in a single model as compared to separate models (see Supplementary Table 5). Thus, separate interaction models were chosen instead of running all interactions simultaneously. To account for alpha error accumulation, we again used a Bonferroni-corrected statistical threshold (α = 5%/4 or 1.25%) to test for significance. Analyses were performed using the lme4 and lmertest package in R (Bates et al., 2014; Kuznetsova et al., 2017).

## Results

Since there are no data on daily embracing behavior in the literature, we first report descriptive data on reported embracing behavior and mood.

## Average Number of Embraces

**Fig. 1:**
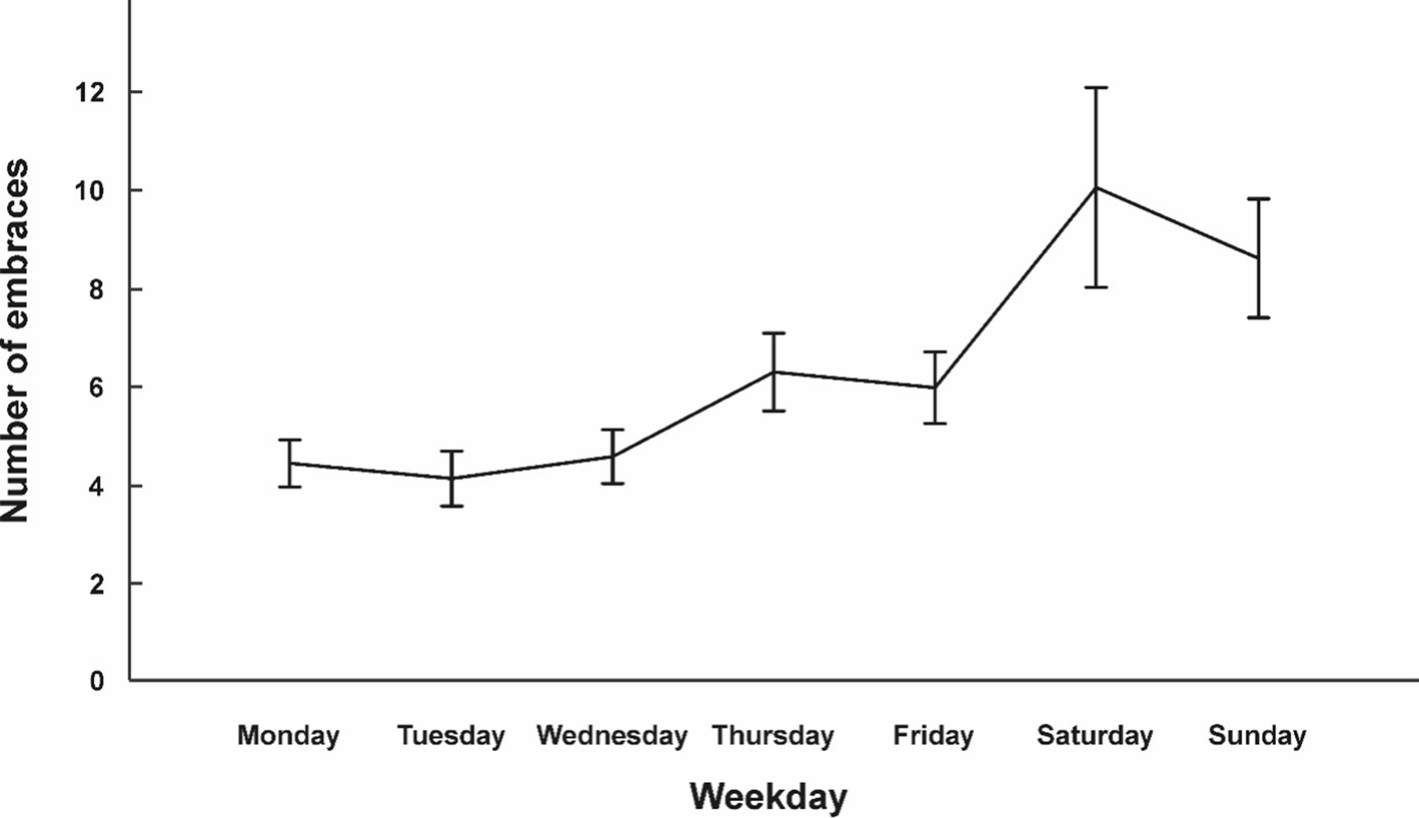
Average number of embraces across the week for all 94 participants. Error bars represent SEM. Individual data points for each participant are shown in Supplementary Fig. 1

On average, participants embraced other people 6.29 times per day (*SD* = 6.15; range 0–150 embraces). To assess whether day of the week was associated with the frequency of embracing, we conducted a repeated-measures ANOVA with day of the week (Monday, Tuesday, Wednesday, Thursday, Friday, Saturday, Sunday) as within-subjects variable (see Fig. 1 for average number of embraces for each day and Table 1 for descriptive statistics). We observed a significant effect of day of the week (*F*_(1.87,174.13)_ = 6.04; *p* = 0.003; *η*_*p*_^*2*^ = 0.06), indicating that embracing frequency changed dependent on the weekday. Bonferroni-corrected post-hoc tests showed that participants embraced significantly more often on Sundays than on Mondays (*p* = 0.022), Tuesdays (*p* = 0.016), and Wednesdays (*p* = 0.005). Despite the absolute larger number of embraces on Saturdays, none of the tests survived correction for multiple comparisons (all *p*s > 0.055). Since the data displayed some significant outliers, we removed all individuals who embraced more than 20 times on any day (*n* = 27) and re-calculated the analysis. The ANOVA showed a comparable effect size (*F*_(4.56,300,92)_ = 4,83; *p* < 0.001; *η*_*p*_^*2*^ = 0.07) indicating that outliers did not influence the result pattern.

**Table 1:** Average values and standard deviations for the number of embraces, number of embraced individuals and mood ratings across the week

Weekday	Monday	Tuesday	Wednesday	Thursday	Friday	Saturday	Sunday	Average
Number of embraces	4.43 ± 0.48	4.12 ± 0.56	4.56 ± 0.55	6.29 ± 0.79	5.97 ± 0.73	10.05 ± 2.04	8.61 ± 1.21	6.29 ± 6.15
Number of embraced individuals	2.39 ± 0.27	2.39 ± 0.34	2.44 ± 0.30	3.70 ± 0.51	3.55 ± 0.41	6.48 ± 1.52	5.29 ± 0.94	3.75 ± 4.26
Daily mood	0.92 ± 0.94	0.99 ± 0.92	0.87 ± 1.07	0.86 ± 1.00	1.01 ± 0.92	1.11 ± 0.91	0.91 ± 1.01	0.95 ± 0.62

## Average Number of Embraced Individuals

**Fig. 2:**
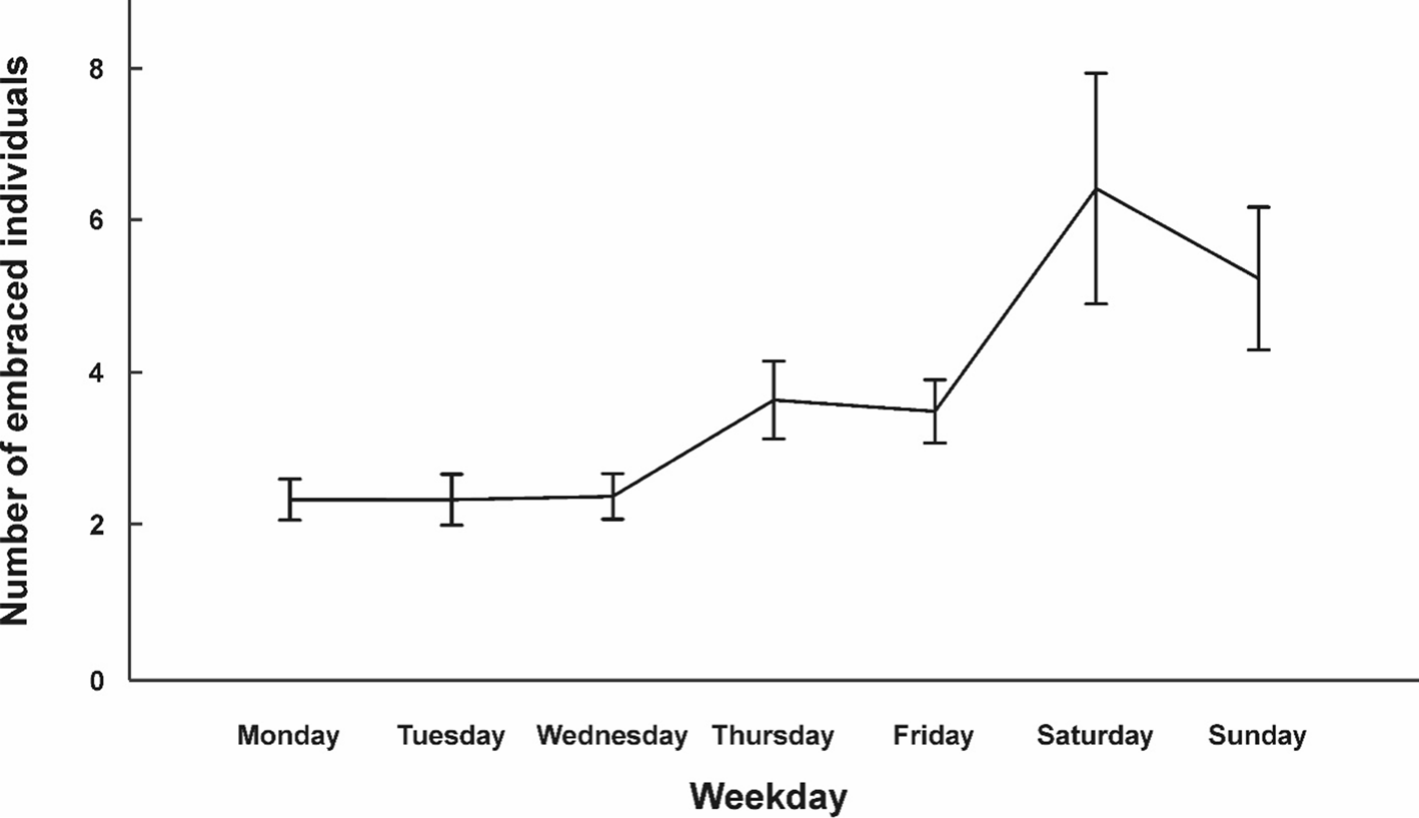
Average number of embraced individuals across the week for all 94 participants. Error bars represent SEM. Individual data points for each participant are shown in Supplementary Fig. 2

In a next step, we examined the number of unique embraced individuals. On average, participants embraced 3.75 unique individuals per day (*SD* = 4.26; range: 0–110 individuals). To assess whether day of the week was associated with this number, we conducted a repeated-measures ANOVA with day of the week as within-subjects variable (see Fig. 2 for average number of embraced individuals for each day and Table 1 for descriptive statistics). We observed a significant effect of day of the week (*F*_(1.62,150.93)_ = 6.04; *p* = 0.005; *η*_*p*_^*2*^ = 0.06), indicating that the number of unique embraced individuals differed with regard to the day of the week. Bonferroni-corrected post-hoc tests showed that participants hugged significantly more individuals on Sundays (mean: 5.29) than on Wednesdays (mean: 2.44, *p* = 0.014). All other post-hoc tests failed to reach significance (all *p*s > 0.057). As for embracing frequency, we removed individuals who embraced more than 20 other people on any day (n = 9) to check whether outliers influenced the result pattern. The ANOVA revealed a slightly lower effect size if outliers were excluded (*F*_(4.94,415.20)_ = 3.45; *p* = 0.005; *η*_*p*_^*2*^ = 0.04). The significant difference between Sundays and Wednesdays disappeared after outlier exclusion (*p* = 0.096). However, excluding outliers resulted in a significant difference between Saturdays and Wednesdays (*p* = 0.012).

The average number of embraces and the average number of unique embraced individuals were strongly correlated (*r*_(93)_ = 0.91; *p* < 0.001), indicating no clear distinction in the underlying measured construct. We therefore decided to only include the absolute number of embraces per day in all further analyses.

## Daily Mood

**Fig. 3:**
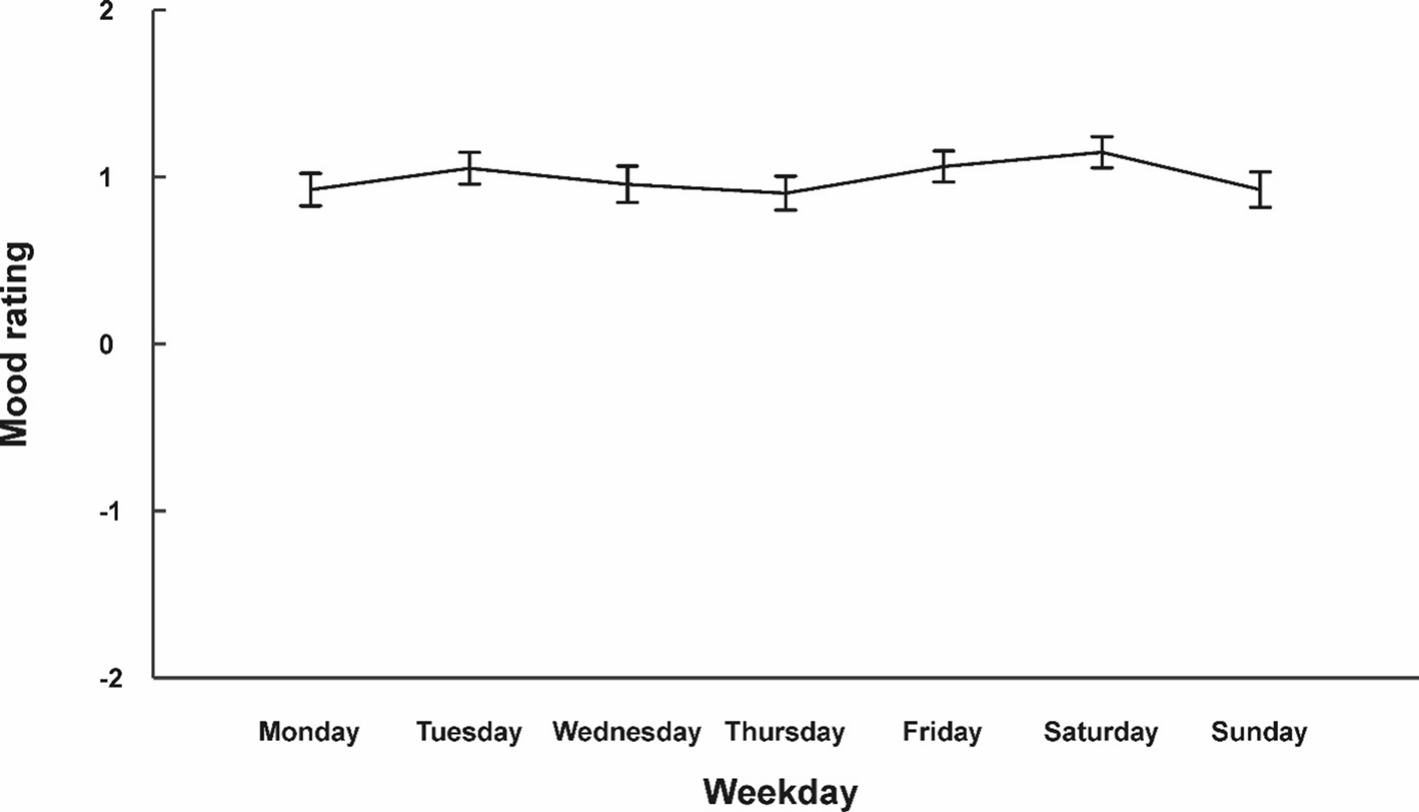
Average mood ratings across the week for all 94 participants. Error bars represent SEM. Individual data points for each participant are shown in Supplementary Fig. 3

On average, participants reported a positive mood rating (0.95 ± 0.62) during the sampled week. To assess whether mood differed across the week, we conducted a repeated-measures ANOVA with day of the week as within-subjects variable (see Fig. 3 for average mood for each day and Table 1 for descriptive statistics). The main effect of day of the week did not reach significance (*F*_(6,558)_ = 1.27; *p* = 0.271), indicating that participants had similar mood ratings across the week.

## Predictors of Daily Mood

To provide detailed insight into the associations between daily mood, daily embracing, personality traits and feelings of loneliness on the individual level, we used a multilevel modeling approach.

In a first analysis, we were interested in the possible factors that are associated with each participant’s daily mood. Here, we included daily embraces as a level 1 predictor. Furthermore, we included feelings of loneliness, Extraversion, Neuroticism, and being in a relationship (relationship vs. single) as level 2 predictors in the analysis. In this model, we found that the daily number of embraces was positively associated with daily mood (*β* = 0.010, *SE* = 0.004, *t* = 2.55, *p* = 0.011). In contrast, Neuroticism (*β* = -0.051, *SE* = 0.025, *t* = 2.04, *p* = 0.044) and loneliness (*β*: − 0.059, *SE* = 0.019, *t* = 3.06, *p* = 0.003) were negatively associated with daily mood. No other predictor reached statistical significance (see Table 2 for details).

**Table 2:** Fixed effects for all predictors without interactions for daily mood

Model 1 (adj*. R*^*2*^ = 0.218)	Regression coefficient	Standard error	95% Confidence interval	t-value	p-value
Intercept	2.126	0.516	[1.131 – 3.121]	4.12	< 0.001***
Daily embraces	0.010	0.004	[0.002 – 0.017]	2.55	0.011*
Loneliness	− 0.530	0.173	[− 0.864 – − 0.196]	3.06	0.003**
Relationship status	0.029	0.118	[− 0.199 – 0.257]	0.25	0.805
Neuroticism	− 0.154	0.075	[− 0.299 – − 0.008]	− 2.04	0.044*
Extraversion	0.075	0.118	[− 0.199 – 0.257]	0.78	0.437

To explore possible interactions of daily embracing with the other predictors, we repeated the analysis and included the cross-level interactions between daily embraces and relationship status, Neuroticism, and Extraversion. We kept all other fixed effects as covariates but investigated the interactions in separate models. In this model, we found an interaction between daily number of embraces and loneliness with respect to daily mood that, however, did not survive the correction for multiple comparisons (*β* = 0.026, *SE* = 0.011, *t* = 2.25, *uncorrected p* = 0.025). Exploring this interaction in more detail revealed that for individuals with low loneliness scores (-1 *SD* from average), the association between daily embraces and momentary mood was not significant (*β* = 0.012, *SE* = 0.006, *t* = 1.98, *p* = 0.118). For individuals with high loneliness scores (+ 1 SD from average), a significant positive association could be identified (*β* = 0.035, *SE* = 0.012, *t* = 2.90, *p* = 0.005, see Supplementary Fig. 4).

We also found a significant interaction prior to correcting for multiple comparisons between daily embraces and Neuroticism (*β* = 0.016, *SE* = 0.007, *t* = 2.47, *uncorrected p* = 0.043). Here, the positive association between daily embraces and momentary mood was higher in individuals with high Neuroticism (+ 1 *SD*, *β* = 0.036, *SE* = 0.012, *t* = 3.17, *p* = 0.002) than in individuals with low Neuroticism (- 1 *SD*, *β* = 0.009, *SE* = 0.004, *t* = 2.44, *p* = 0.015, see Supplementary Fig. 5).

Finally, prior to correction, the interaction between daily embraces and relationship status reached significance (*β* = 0.025, *SE* = 0.013, *t* = 1.98, *uncorrected p* = 0.048, see Supplementary Fig. 6). We observed a significant positive association between daily embraces and momentary mood in singles (*β* = 0.032, *SE* = 0.012, *t* = 2.68, *p* = 0.008) but not in individuals in a relationship (*β* = 0.007, *SE* = 0.004, *t* = 1.81, *p* = 0.071).

Regression coefficients, standard errors, and confidence intervals for all predictors of each model are depicted in Supplementary Tables 6—9.

## Predictors of Life Satisfaction

Life satisfaction was assessed only once and could therefore not be examined on the daily level. Instead, we ran a single level linear regression with life satisfaction as dependent variable and the average number of embraces, loneliness, relationship status (relationship vs. single), Neuroticism, and Extraversion as predictors. This first model revealed a negative association of loneliness with life satisfaction (β = − 0.377, *SE* = 0.148, *t* = 2.56, *p* = 0.012). Importantly, average daily embraces were not significantly associated with life satisfaction. Model details are depicted in Table 3.

**Table 3:** Fixed effects for all predictors without interactions for life satisfaction

Model 1 (adj*. R*^*2*^ = 0.366)	Regression coefficient	Standard error	95% Confidence interval	t-value	p-value
Intercept	44–484	4.071	[25.393 – 41.575]	8.23	< 0.001***
Loneliness	− 3.394	1.328	[− 6.033 –0.756]	2.56	0.012*
Relationship status	− 1.787	0.910	[− 3.596 − 0.021]	1.96	0.053
Neuroticism	− 1.132	0.582	[− 2.288–0.024]	1.95	0.055
Extraversion	0.803	0.732	[− 0.652 – 2.257]	1.10	0.276
Average embraces	0.124	0.078	[− 0.031 – 0.278]	1.59	0.115

We then explored the interactions between the average number of embraces and the remaining predictors. Identically to the mood analysis, we tested the interactions in separate models.

**Fig. 4:**
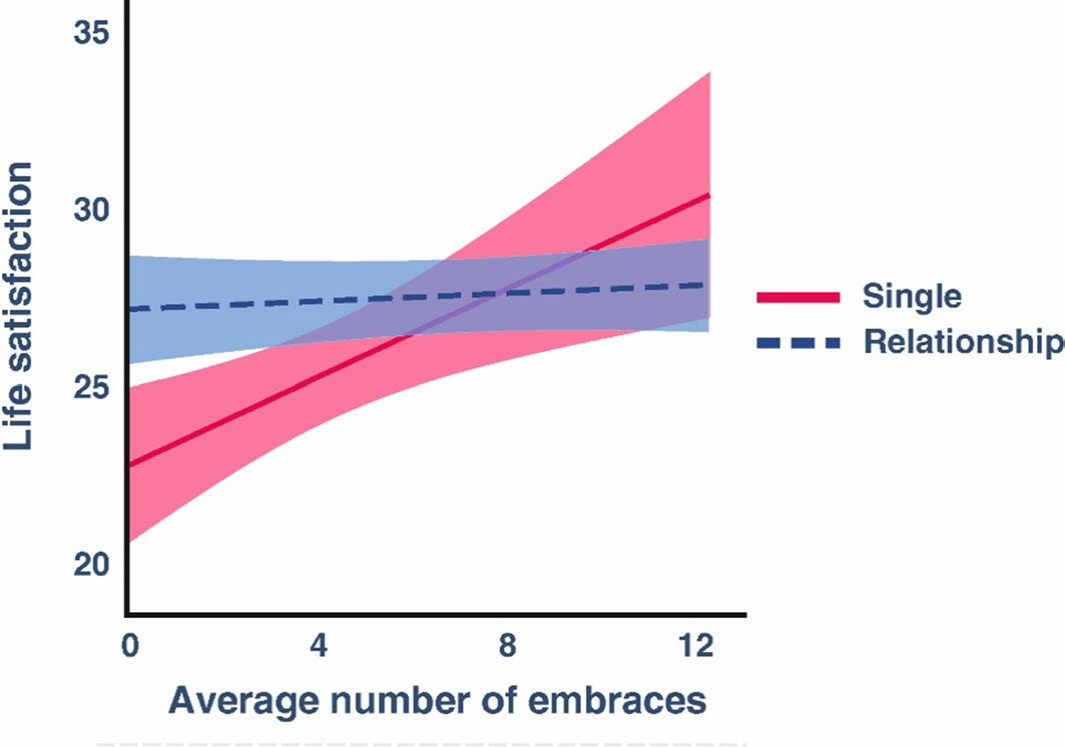
Interaction between average embracing frequency and relationship status on life satisfaction ratings. Shaded areas represent the 95% confidence interval

The only interaction that reached significance after correction was between the average number of embraces and relationship status. We found a significant positive interaction between these factors (*β* = 0.564, *SE* = 0.212, *t* = 2.66, *p* = 0.009). Investigating this interaction in more detail revealed a weak association for individuals in a relationship (*β* = 0.056, *SE* = 0.079, *p* = 0.483) but a strong association for singles (*β* = 0.619, *SE* = 0.201, *p* = 0.003), suggesting that singles benefit more strongly from embracing than individuals in a relationship (Fig. 4). Regression coefficients, standard errors, and confidence intervals for all predictors of each model are depicted in Supplementary Tables 10—13.

## Discussion

The aim of the present study was to provide descriptive reference data on changes in daily embracing behavior and to elucidate the associations between daily mood as well as life satisfaction and trait variables such as loneliness, personality variables, or relationship status.

Analysis of the EMA data that were collected before the COVID-19 pandemic revealed that on average, participants in our German sample embraced other people about six times a day. These six embraces were divided across an average of about four different persons per day. Both numbers were below their respective averages on weekdays and above their respective averages on weekends. This effect is likely caused by differences in activity composition between weekends and weekdays before the COVID-19 pandemic. For example, participants might be more likely to meet with their friends and families on weekends. These are situations in which embraces occur more commonly than at work or at the university. Similar to our observation of embracing differences over the week, cortisol awakening responses have also been demonstrated to show clear weekday-weekend differences with significantly lower cortisol secretion on weekends (Schlotz et al., 2004). While these results were primarily interpreted in the context of work stress, fewer social touch interactions could also have been a contributing factor as these have been demonstrated to decrease cortisol levels (Sumioka et al., 2013).

Using multilevel modeling, we could get insight into the association between daily mood and daily embracing at the within-person level. Here, daily mood was positively associated with higher embracing frequency. We could thus overall replicate the association reported by Murphy et al. (2018) who found that embracing frequency is associated with increases in momentary mood, especially in conflict situations. With respect to loneliness, we could first of all replicate the well-known findings from the literature that higher levels of loneliness are associated with decreases in mood and life satisfaction (Goodwin et al., 2001; Zawadzki et al., 2013). Prior to correction, we also found a significant interaction between daily embraces and loneliness. Here, the negative effects of loneliness on momentary mood were reduced when the individual reported more daily hugs. These results are supporting the idea that negative mood associated with loneliness can be relieved through means of social touch in line with findings from Heatley Tejada et al. (2020) who found that social touch can relieve of feelings of loneliness directly. In contrast, there was no significant effect of embracing on daily mood in individuals with low loneliness scores suggesting that their daily mood is not associated with this form of social touch. It should be noted that the non-significant association was positive nonetheless, indicating that the embraces also benefit individuals that do not feel lonely, albeit to a much lesser extent. The interpretation of this interaction needs to be treated carefully, however, due to the exploratory nature of this finding.

In contrast to the results for the association between daily mood and daily embraces, we found no evidence that higher tendencies to engage in social touch operationalized through the average number of embraces were associated with increased life satisfaction. While we could thus not find a simple effect between average embracing and life satisfaction, there was however a significant interaction between the tendency to embrace and relationship status. Here, singles demonstrated a much stronger benefit from embracing over the week compared to individuals in a relationship. Since a similar trend was observed also for momentary mood, it seems that the beneficial effects of embraces are moderated by the relationship status, a phenomenon previously observed also in the context of the stress-buffering effects of embraces (Pauley et al., 2015).

For personality traits, we could find a significant negative association between Neuroticism and daily mood, and a trend towards significance for life satisfaction in accordance with several other studies and meta-analyses (Anglim et al., 2020; Larsen & Ketelaar, 1989; Ruiz-Caballero & Bermúdez, 1995; Schimmack et al., 2004). Similar to our findings for loneliness, Neuroticism was also indicated to moderate the relationship between daily embraces and momentary mood, as individuals demonstrating higher scores of Neuroticism benefitted more strongly from a large number of embraces. Since this analysis did not survive correction for multiple comparisons, this result has to be treated with caution however. For Extraversion, we surprisingly found no positive association with daily mood or life satisfaction. A possible explanation for this discrepancy from the literature could be derived from studies that found that the link between Extraversion and positive affect is mediated by social interactions and, if controlled for, Extraversion does not correlate with positive mood (Srivastava et al., 2008). Since we did not assess the number of social interactions directly, this interpretation however remains rather speculative.

For all of the above-mentioned results, we need to stress that they are correlational. Therefore, we cannot causally infer if embracing leads to an increase in positive mood or life satisfaction and vice versa. Additionally, it cannot be ruled out that, for example, lonely individuals who were already in a more positive mood for other reasons tended to embrace more often—or that they were already in a negative mood and thus refrained from making social contacts with other people, reducing the number of embraces. Furthermore, other variables that strongly correlate with the number of embraces, such as increases in social encounters in general, could have caused the increase in overall mood. While we did not assess this in more detail to facilitate the data collection process, this is, however, less likely since the study of Murphy et al. (2018) did not find an effect of social encounters per se. Given the strong evidence of embracing on health-related aspects, it indicates that embraces could have mediated the increase in positive mood as well. It is critical to experimentally assess this link in the future. Here, studies could experimentally assess the relation of embracing and mood in a systematic way, e.g., by putting participants experimentally in a bad mood and then compare to what extent embracing enhances mood compared to a non-embracing control group, similar to the design of the above-mentioned embracing and stress study (Grewen et al., 2003). A further limitation of the present study is related to the assessment of embraces in the questionnaires. We did not explicitly ask participants to rate the intimacy and pleasantness of the embrace. Recent research has, however, suggested that especially intimate embraces are associated with a reduction in, for example, anxiety and overall mental health (Mohr et al., 2021). Thus, the overall effect for daily embracing on momentary mood could have been diluted due to the abundance of friendly and even professional embraces. Finally, it is possible that participants behaved differently than usual due to the nature of being “observed” since they had to report their behavior every day. This concept of reactivity has however been suggested to be of low effect in ambulatory assessment research (Barta et al., 2012; Macintyre et al., 2021;).

This is the first smartphone-based EMA study on embracing, and we are convinced that this technique has great potential to further elucidate the role of embracing in social interactions and for mental and physical well-being as both these parameters represent highly dynamic constructs that are influenced by both state and trait variables. Several potential follow-up studies to our present work come to mind:

Recently, the importance of assessing social touch not only in Western populations, but also in non-W.E.I.R.D. (Western, Educated, Industrialized, Rich, and Democratic) cohorts has been highlighted for kissing, another form of social touch (Karim et al., 2017). Future studies also should investigate the links between embracing and loneliness in a broader variety of samples from different countries. This is essential in order to assess the influences of societal learning or cultural norms on embracing and kissing (Henrich et al., 2010).

Another variable that has been discussed to be important in the context of social touch is whether the investigated individual initiated the embrace or received it. This variable has recently also been discussed in the context of kissing (Karim et al., 2017), where strong sex differences were demonstrated (males initiated the kiss significantly more often than females). For embracing, it might be speculated that individuals in a positive mood are more likely to initiate embraces themselves to convey positive emotions or social support. In contrast, individuals in a bad mood might be more likely to receive embraces, as their partners or friends might embrace them to comfort or solace them. This relation between mood and embracing might also be relevant in the context of a more systematic investigation of the reasons why humans embrace.

Furthermore, EMA could also be used to investigate other forms of social touch than embracing like for example kissing (Ocklenburg & Güntürkün, 2009) or cradling (Forrester & Todd, 2018; Forrester et al., 2018; Packheiser et al., 2020). In this regard, it should also be mentioned that social touch is not limited to human–human interactions but can also occur in human-pet interactions. For example, a recent study investigated cradling in human–dog interactions and found that humans have a preference to cradle their dogs on the left (Abel, 2010), a result that has been attributed to a stronger emotional bond for the cradling of human children (Packheiser et al., 2019). Future studies could investigate whether hugging or cradling a pet has similar associations with loneliness than embracing a human has.

Finally, we want to highlight that our data was collected prior to the onset of the COVID-19 pandemic. Since levels of loneliness were substantially increased during the pandemic, especially in lockdown situations (Groarke et al., 2020; Killgore et al., 2020), this could have also potentiated the role of embracing to alleviate it. Future research is however needed to identify how the pandemic impacted the role of social touch in our lives.

## Supplementary Information

Below is the link to the electronic supplementary material.

Supplementary file1 (DOCX 730 KB)

## Data Availability

Data and code will be made available upon request.
