# Supplementary material for: The Association of Embracing with Daily Mood and General Life Satisfaction: An Ecological Momentary Assessment Study
Source: J Nonverbal Behav. 2022 Aug 4;46(4):519–36. doi: 10.1007/s10919-022-00411-8 (PMC9362016; doi:10.1007/s10919-022-00411-8)
Supplement: Supplementary file 1 — Supplementary file1 (DOCX 730 KB) [file 10919_2022_411_MOESM1_ESM.docx]

Supplemental Appendix


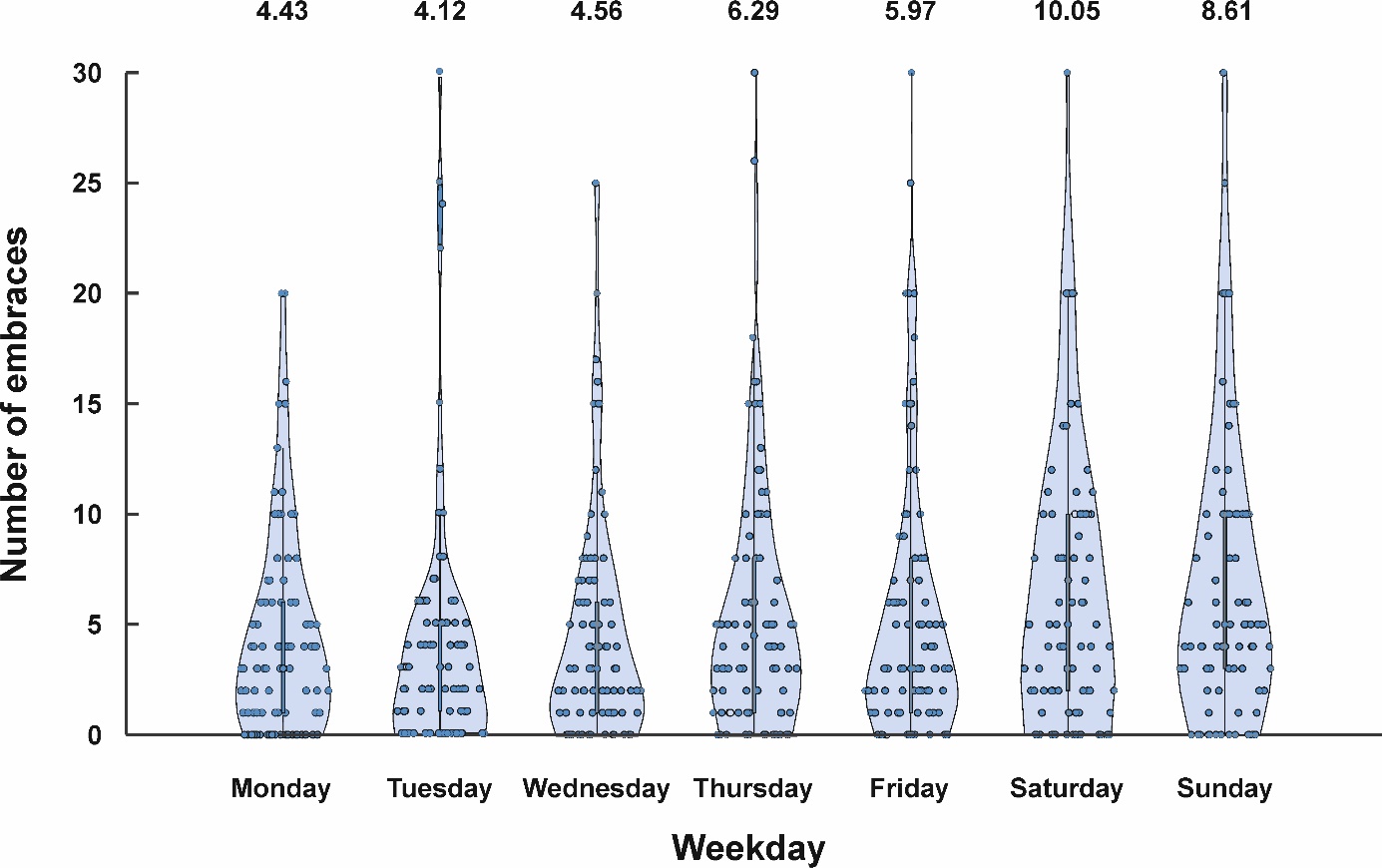


Supplementary Figure 1. Violin plot for number of embraces across the week for all 94 participants. Numbers above each violin plot reflect the average number of embraces for a given weekday. Potential outliers are included in the figure.


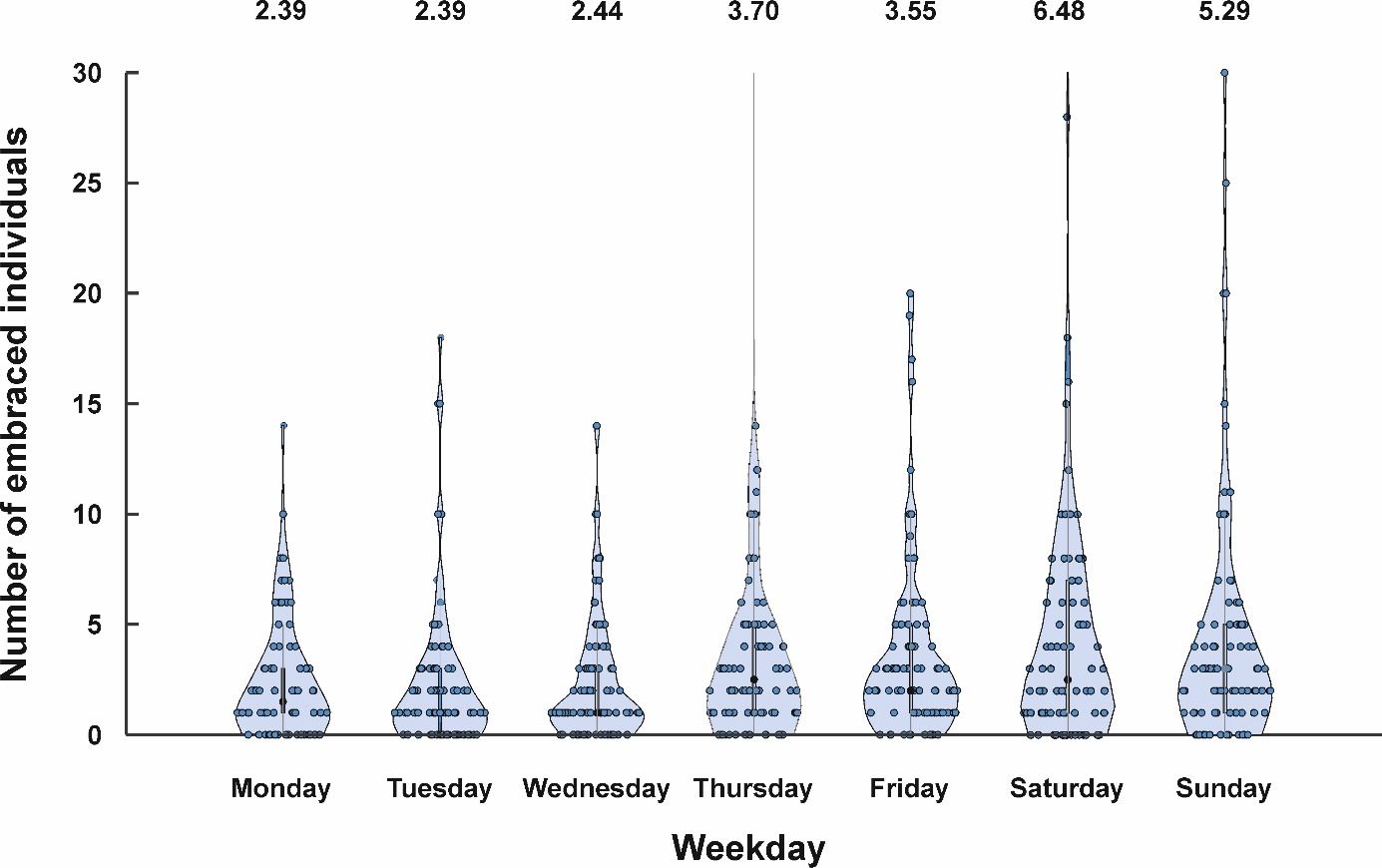


Supplementary Figure 2. Violin plot for number of embraced individuals across the week for all 94 participants. Numbers above each violin plot reflect the average number of embraced individuals for a given weekday. Potential outliers are included in the figure.


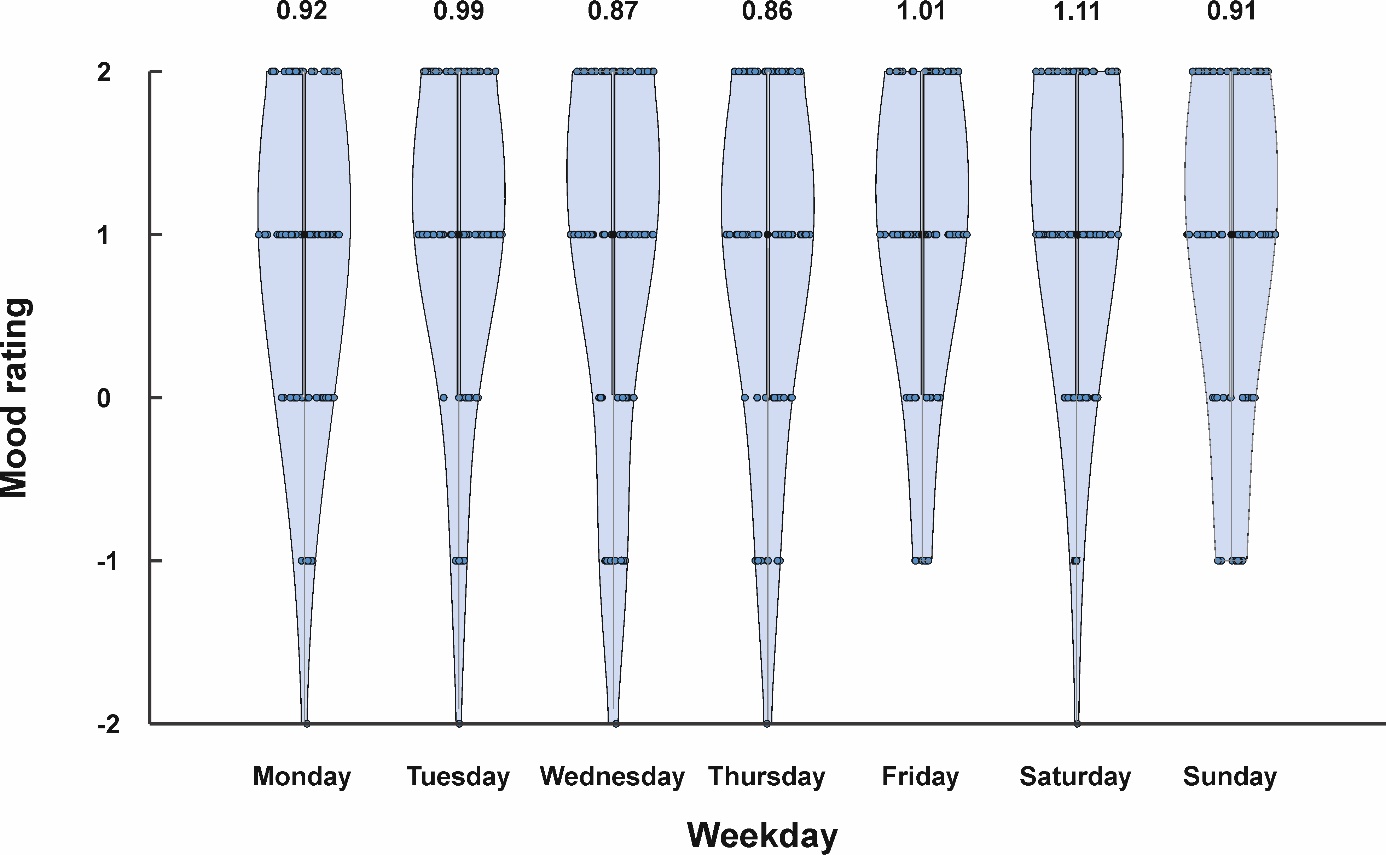


Supplementary Figure 3. Violin plot for number of mood ratings across the week for all 94 participants. Numbers above each violin plot reflect the average mood ratings for a given weekday.


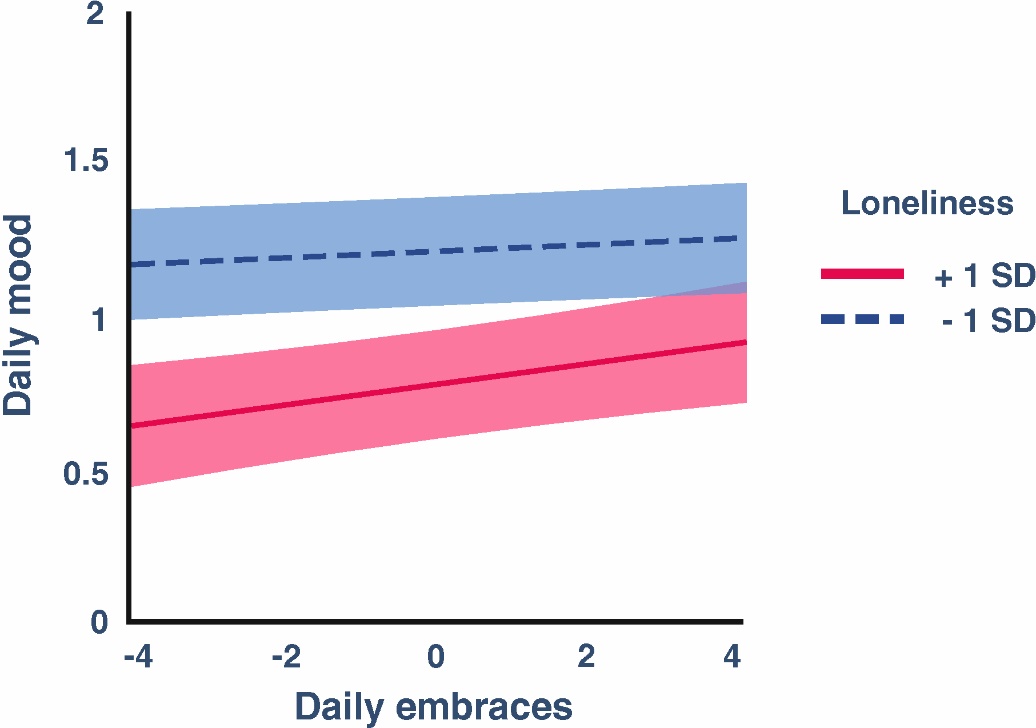


Supplementary Figure 4. Interaction between daily embracing frequency and loneliness on daily mood. Shaded areas represent the 95% confidence interval. Note that daily embraces are plotted centered to the person mean.


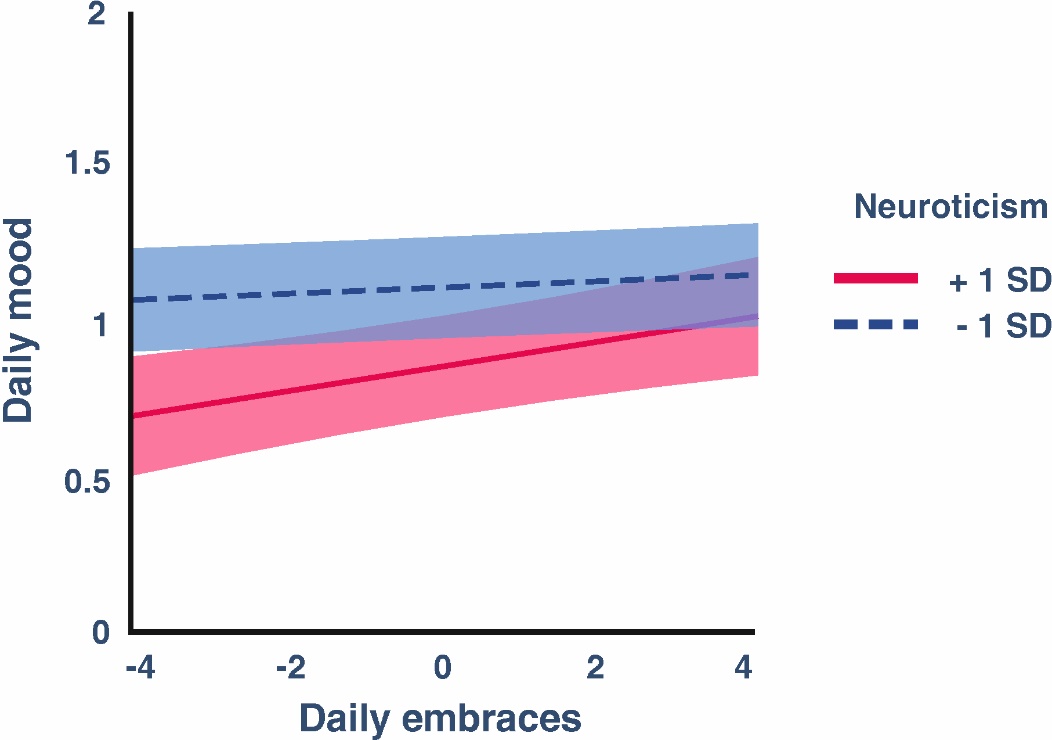


Supplementary Figure 5. Interaction between daily embracing frequency and Neuroticism on daily mood. Shaded areas represent the 95% confidence interval. Note that daily embraces are plotted centered to the person mean.


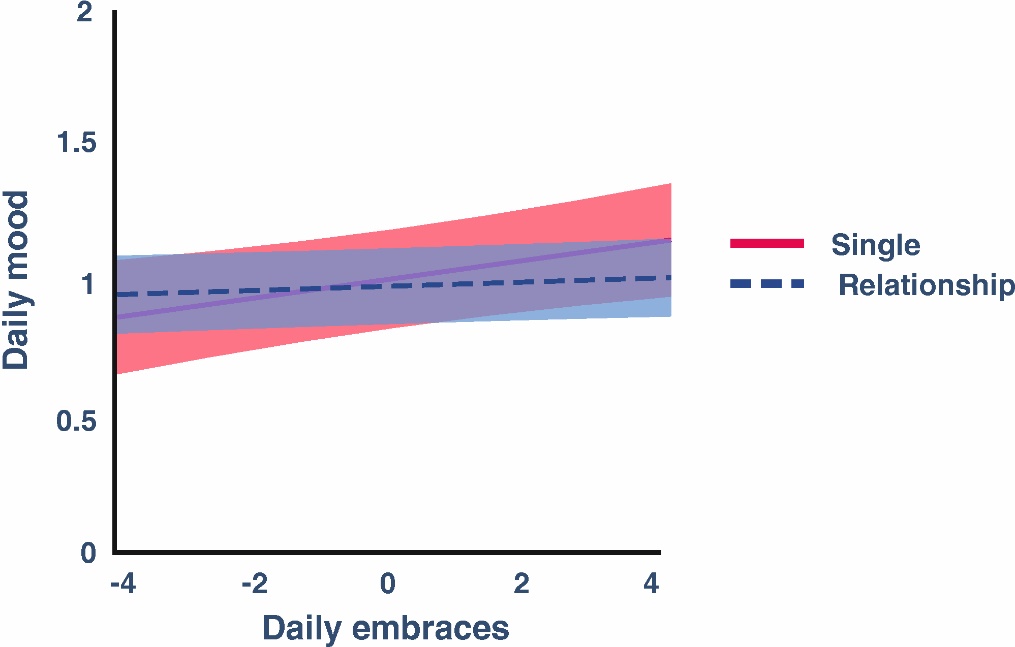


Supplementary Figure 6. Interaction between daily embracing frequency and relationship status on daily mood. Shaded areas represent the 95% confidence interval. Note that daily embraces are plotted centered to the person mean.

**Supplementary Table 1.** Variance inflation factors (VIF) between the independent variables for daily mood as dependent variable for the base model.

|  | Daily embraces | Loneliness | Relationship status | Neuroticism | Extraversion |
| --- | --- | --- | --- | --- | --- |
| VIF Model 1 | 1.00 | 1.61 | 1.04 | 1.25 | 1.33 |

**Supplementary Table 2.** Variance inflation factors (VIF) between the independent variables for daily mood as dependent variable for the cross-level interaction models. Models 2 to 5 refer to the separate cross-level interaction models and model 6 refers to the model containing all cross-level interactions simultaneously.

|  | | Daily em-braces | Loneli-ness | Relationship status | Neuro-ticism | Extra-version | Daily embraces * Loneliness | Daily embraces * Relationship status | Daily embraces * Neuroticism | Daily embraces * Extra-version | |
| --- | --- | --- | --- | --- | --- | --- | --- | --- | --- | --- | --- |
| VIF Model 2 | | 18.45 | 1.61 | 1.04 | 1.25 | 1.33 | 18.45 | - | - | - |  |
|  | |  |  |  |  |  |  |  |  |  |  |
| VIF Model 3 | | 1.11 | 1.61 | 1.04 | 1.25 | 1.33 | - | 1.11 | - | - |  |
| VIF Model 4 | | 13.67 | 1.61 | 1.04 | 1.25 | 1.33 | - | - | 13.67 | - |  |
| VIF Model 5 | | 43.83 | 1.61 | 1.04 | 1.25 | 1.33 | - | - | - | 43.83 |  |
| VIF Model 6 | 160.26 | | 1.61 | 1.04 | 1.25 | 1.33 | 33.36 | 1.45 | 24.70 | 72.55 |  |

**Supplementary Table 3.** Differences in all other level 2 predictors and life satisfaction between singles and individuals in a relationship as measured via an independent sample t-test. Errors represent SDs.

|  | Single | Relationship | t-value | p-value |
| --- | --- | --- | --- | --- |
| Loneliness | 1.89  ± 0.39 | 1.73  ± 0.42 | 1.86 | .066 |
| Extraversion  Neuroticism  Average embraces  Life satisfaction | 3.04  ± 0.68  2.93  ± 0.82  4.28  ± 3.49  24.94  ± 4.84 | 3.21  ± 0.67  2.73  ± 0.84  7.48  ± 7.06  28.03  ± 4.78 | 1.19  1.16  2.95  3.01 | .237  .251  .004**  .004** |

**Supplementary Table 4.** Variance inflation factors (VIF) between the independent variables for life satisfaction as dependent variable for the base model.

|  | Average embraces | Loneliness | Relationship status | Neuroticism | Extraversion |
| --- | --- | --- | --- | --- | --- |
| VIF Model 1 | 1.26 | 1.66 | 1.08 | 1.31 | 1.33 |

**v**Variance inflation factors (VIF) between the independent variables for life satisfaction as dependent variable for the interaction models. Models 2 to 5 refer to the separate interaction models and model 6 refers to the model containing all interactions simultaneously.

|  | | Avg. em-braces | Loneli-ness | Relationship status | Neuro-ticism | Extra-version | Avg. embraces * Loneliness | Avg. embraces * Relationship status | Avg. embraces * Neuroticism | Avg. embraces * Extra-version | |
| --- | --- | --- | --- | --- | --- | --- | --- | --- | --- | --- | --- |
| VIF Model 2 | | 14.97 | 2.39 | 1.08 | 1.31 | 1.35 | 13.45 | - | - | - |  |
|  | |  |  |  |  |  |  |  |  |  |  |
| VIF Model 3 | | 1.41 | 1.66 | 2.45 | 1.31 | 1.34 | - | 2.29 | - | - |  |
| VIF Model 4 | | 12.38 | 1.69 | 1.09 | 2.29 | 1.34 | - | - | 11.18 | - |  |
| VIF Model 5 | | 38.76 | 1.74 | 1.09 | 1.31 | 2.37 | - | - | - | 40.46 |  |
| VIF Model 6 | 151.44 | | 4.08 | 3.21 | 3.51 | 3.45 | 30.84 | 3.32 | 22.42 | 74.88 |  |

**Supplementary Table 6.** Fixed effects for all predictors and the interaction between daily embraces and loneliness for daily mood. The corrected significance threshold is *p* = 0.0125.

| Model 2  (adj*. R²* = 0.225) | Regression coefficient | Standard error | 95% Confidence Interval | t-value | p-value |
| --- | --- | --- | --- | --- | --- |
| Intercept | 2.126 | 0.516 | [1.131 – 3.120] | 4.12 | < 0.001*** |
| Daily embraces | -0.025 | 0.016 | [-0.057 – 0.006] | 1.59 | 0.113 |
| Loneliness | -0.530 | 0.173 | [-0.863 – -0.196] | 3.06 | 0.003** |
| Relationship status | 0.029 | 0.118 | [-0.199 – 0.257] | 0.25 | 0.805 |
| Neuroticism | -0.153 | 0.075 | [-0.299 – -0.008] | 2.04 | 0.044* |
| Extraversion | 0.076 | 0.097 | [-0.111 – 0.262] | 0.78 | 0.437 |
| Daily embraces * Loneliness | 0.026 | 0.011 | [0.003 – 0.048] | 2.25 | 0.025(*) |

**Supplementary Table 7.** Fixed effects for all predictors and the interaction between daily embraces and relationship status for daily mood. The corrected significance threshold is *p* = 0.0125.

| Model 3  (adj*. R²* = 0.223) | Regression coefficient | Standard error | 95% Confidence Interval | t-value | p-value |
| --- | --- | --- | --- | --- | --- |
| Intercept | 2.126 | 0.516 | [1.420 – 4.071] | 4.12 | < 0.001*** |
| Daily embraces | 0.007 | 0.004 | [-0.001 – 0.014] | 1.81 | 0.070 |
| Loneliness | -0.530 | 0.173 | [-0.863 – -0.196] | 3.06 | 0.003** |
| Relationship status | 0.029 | 0.118 | [-0.199 – 0.257] | 0.25 | 0.805 |
| Neuroticism | -0.153 | 0.075 | [-0.299 – -0.008] | 2.04 | 0.044* |
| Extraversion | 0.076 | 0.097 | [-0.111 – 0.262] | 0.78 | 0.437 |
| Daily embraces * relationship status | 0.025 | 0.013 | [0.0003 – 0.049] | 1.98 | 0.048(*) |

**Supplementary Table 8.** Fixed effects for all predictors and the interaction between daily embraces and Neuroticism for daily mood. The corrected significance threshold is *p* = 0.0125.

| Model 4  (adj*. R²* = 0.226) | Regression coefficient | Standard error | 95% Confidence Interval | t-value | p-value |
| --- | --- | --- | --- | --- | --- |
| Intercept | 2.126 | 0.516 | [1.420 – 4.071] | 4.12 | < 0.001*** |
| Daily embraces | - 0.023 | 0.014 | [-0.115 – 0.002] | -1.25 | 0.213 |
| Loneliness | -0.530 | 0.173 | [-0.863 – -0.196] | 3.06 | 0.003** |
| Relationship status | 0.029 | 0.118 | [-0.199 – 0.257] | 0.25 | 0.805 |
| Neuroticism | -0.153 | 0.075 | [-0.299 – -0.008] | 2.04 | 0.044* |
| Extraversion | 0.076 | 0.097 | [-0.111 – 0.262] | 0.78 | 0.437 |
| Daily embraces * Neuroticism | 0.005 | 0.002 | [0.001 – 0.010] | 2.47 | 0.043(*) |

**Supplementary Table 9.** Fixed effects for all predictors and the interaction between daily embraces and Extraversion for daily mood. The corrected significance threshold is *p* = 0.0125.

| Model 5  (adj*. R²* = 0.225) | Regression coefficient | Standard error | 95% Confidence Interval | t-value | p-value |
| --- | --- | --- | --- | --- | --- |
| Intercept | 2.126 | 0.516 | [1.420 – 4.071] | 4.12 | < 0.001*** |
| Daily embraces | 0.054 | 0.025 | [0.006– 0.103] | 2.21 | 0.027* |
| Loneliness | -0.530 | 0.173 | [-0.863 – -0.196] | 3.06 | 0.003** |
| Relationship status | 0.029 | 0.118 | [-0.199 – 0.257] | 0.25 | 0.805 |
| Neuroticism | -0.153 | 0.075 | [-0.299 – -0.008] | 2.04 | 0.044* |
| Extraversion | 0.076 | 0.097 | [-0.111 – 0.262] | 0.78 | 0.437 |
| Daily embraces * Extraversion | -0.012 | 0.006 | [-0.025 – 0.001] | 1.84 | 0.063 |

**Supplementary Table 10.** Fixed effects for all predictors and the interaction between average embracing and loneliness for life satisfaction. The corrected significance threshold is *p* = 0.0125.

| Model 2  (adj*. R²* = 0.384) | Regression coefficient | Standard error | 95% Confidence Interval | t-value | p-value |
| --- | --- | --- | --- | --- | --- |
| Intercept | 35.679 | 4.26 | [31.511 – 55.070] | 8.36 | < 0.001*** |
| Average embraces | -0.280 | 0.265 | [-0.807- 0.247] | 1.06 | 0.294 |
| Loneliness | -4.783 | 1.580 | [-7.923 - -1.643] | 3.03 | 0.003** |
| Relationship status | -1.766 | 0.903 | [-3.560- 0.028] | 1.96 | 0.054 |
| Neuroticism | -1.057 | 0.579 | [-2.207 – 0.093] | 1.83 | 0.071 |
| Extraversion | 0.695 | 0.729 | [-0.754- 2.143] | 0.95 | 0.343 |
| Loneliness * average embraces | 0.285 | 0.020 | [-0.071 – 0.642] | 1.59 | 0.116 |

**Supplementary Table 11.** Fixed effects for all predictors and the interaction between average embracing and relationship status for life satisfaction. The corrected significance threshold is *p* = 0.0125.

| Model 3  (adj*. R²* = 0.414) | Regression coefficient | Standard error | 95% Confidence Interval | t-value | p-value |
| --- | --- | --- | --- | --- | --- |
| Intercept | 34.649 | 3.962 | [26.774 – 42.524] | 8.75 | < 0.001*** |
| Loneliness | -3.586 | 1.286 | [-6.142 - -1.030] | 2.79 | 0.006** |
| Relationship status | -4.418 | 1.325 | [-7.051 - -1.785] | 3.34 | 0.001** |
| Neuroticism | -1.086 | 0.562 | [-2.205 – 0.033] | 1.93 | 0.057 |
| Extraversion | 0.662 | 0.710 | [-0.750 - 2.073] | 0.93 | 0.354 |
| Average embraces | -0.508 | 0.249 | [-1.003 - -0.013] | 0.704 | 0.483 |
| Relationship status * average embraces | 0.564 | 0.212 | [0.142 - 0.985] | 2.66 | 0.009** |

**Supplementary Table 12.** Fixed effects for all predictors and the interaction between average embracing and Neuroticism for life satisfaction. The corrected significance threshold is *p* = 0.0125.

| Model 4  (adj*. R²* = 0.379) | Regression coefficient | Standard error | 95% Confidence Interval | t-value | p-value |
| --- | --- | --- | --- | --- | --- |
| Intercept | 34.301 | 4.097 | [26.159 – 42.444] | 8.37 | < 0.001*** |
| Loneliness | -3.161 | 1.333 | [-5.809 - -0.512] | 2.37 | 0.020* |
| Relationship status | -1.705 | 0.908 | [-3.510 – 0.100] | 1.88 | 0.064 |
| Neuroticism | -1.814 | 0.767 | [-3.339 - -0.289] | 2.36 | 0.020* |
| Extraversion | 0.285 | 0.243 | [-0.595 – 2.305] | 1.17 | 0.245 |
| Average embraces | -0.187 | 0.187 | [-0.669 – 0.294] | 0.77 | 0.441 |
| Neuroticism * average embraces | 0.153 | 0.113 | [-0.072 - 0.378] | 1.36 | 0.134 |

**Supplementary Table 13.** Fixed effects for all predictors and the interaction between average embracing and Extraversion for life satisfaction. The corrected significance threshold is *p* = 0.0125.

| Model 5  (adj*. R²* = 0.393) | Regression coefficient | Standard error | 95% Confidence Interval | t-value | p-value |
| --- | --- | --- | --- | --- | --- |
| Intercept | 27.765 | 4.934 | [17.958 – 37-571] | 5.63 | < 0.001*** |
| Loneliness | -2.808 | 1.339 | [-5.469 - -0.146] | 2.10 | 0.039* |
| Relationship status | -1.590 | 0.901 | [-3.381 – 0.201] | 1.76 | 0.081 |
| Neuroticism | -1.119 | 0.572 | [-2.257 – 0.018] | 1.96 | 0.054 |
| Extraversion | 2.065 | 0.961 | [0.155 – 3.974] | 2.24 | 0.027* |
| Average embraces | 0.951 | 0.320 | [0.155 – 1.702] | 2.15 | 0.034* |
| Extraversion * average embraces | -0.223 | 0.112 | [-0.148 – 0.0003] | 1.99 | 0.050 |
